# Improved Efficiency and Lesion Detection in Small Bowel Capsule Endoscopy Using the Open‐Source Artificial Intelligence Model SEE‐AI

**DOI:** 10.1002/deo2.70346

**Published:** 2026-05-15

**Authors:** Satoshi Miyazono, Junji Umeno, Tomohiro Nagasue, Takuto Saiki, Hisamitsu Kaku, Takehiro Torisu, Akihito Yokote, Keisuke Kawasaki, Yutaro Ihara, Yuichi Matsuno, Noriyuki Imazu, Tomohiko Moriyama, Ahmed Nashaat Mohamed, Katsuya Hirakawa, Hajime Yamagata, Yasuharu Okamoto, Koichi Kurahara, Shinichiro Yada, Akira Harada, Tetsuro Ago

**Affiliations:** ^1^ Department of Medicine and Clinical Science Graduate School of Medical Sciences Kyushu University Fukuoka Japan; ^2^ Department of Endoscopic Diagnostics and Therapeutics Kyushu University Hospital Fukuoka Japan; ^3^ International Medical Department Kyushu University Hospital Fukuoka Japan; ^4^ The National Hepatology and Tropical Medicine Research Institute Cairo Egypt; ^5^ Division of Gastroenterology Fukuoka Red Cross Hospital Fukuoka Japan; ^6^ Department of Gastroenterology Hamanomachi Hospital Fukuoka Japan; ^7^ Department of Gastroenterology Kyushu Central Hospital of the Mutual Aid Association of Public School Teachers Fukuoka Japan; ^8^ Division of Gastroenterology Matsuyama Red Cross Hospital Ehime Japan; ^9^ Department of Gastroenterology Onga Nakama Medical Association, Onga Hospital Fukuoka Japan; ^10^ Division of Gastroenterology Yamaguchi Red Cross Hospital Yamaguchi Japan

**Keywords:** artificial intelligence, capsule endoscopy, gastrointestinal tract, small intestine, suspected small‐bowel bleeding

## Abstract

**Objectives:**

Small bowel capsule endoscopy (CE) produces lengthy videos that are time‐consuming to review and susceptible to missed lesions. We evaluated whether an open‐source, pretrained artificial intelligence (AI) model (SEE‐AI) could improve diagnostic performance and interpretation efficiency compared with conventional reading.

**Methods:**

We retrospectively analyzed 249 PillCam SB3 examinations performed between 2007 and 2022 at six hospitals, using a two‐reader crossover design. SEE‐AI (confidence threshold 0.1) generated annotated videos with bounding boxes for eight lesion categories. The primary endpoints were sensitivity for lesion detection on a per‐lesion and per‐patient basis. Secondary endpoints included specificity, predictive values, overall accuracy, and reading time. A prespecified subgroup analysis evaluated cases of suspected small‐bowel bleeding (SSBB), focusing on Saurin P1+P2 hemorrhagic lesions.

**Results:**

Across 1550 adjudicated lesions, AI‐assisted reading demonstrated higher sensitivity than conventional reading (per‐lesion: 98.8% [1532/1550] vs. 86.4% [1339/1550]; per‐patient: 99.1% [464/468] vs. 80.3% [376/468]; both *p* < 0.0001). The mean reading time decreased from 17.9 to 13.7 min (*p* < 0.0001). In SSBB cases (*n* = 131), sensitivity for P1+P2 lesions improved on both a per‐lesion basis (98.2% [439/447] vs. 82.8% [370/447]) and per‐patient basis (98.6% [145/147] vs. 73.5% [108/147]), with a shorter reading time (14.1 vs. 18.0 min; all *p* < 0.0001).

**Conclusions:**

In this multicenter evaluation, SEE‐AI significantly improved lesion detection and reduced reading time for CE interpretation, including SSBB cases, while maintaining openness and reproducibility. AI‐assisted reading may reduce clinicians’ workload and support the adoption of SEE‐AI as a practical tool ― and a potential future standard of care ― for small bowel CE.

**Trial Registration:**

N/A.

## Graphical abstract

AI‐assisted reading with the open‐source SEE‐AI model improves lesion detection sensitivity and reduces reading time in small‐bowel capsule endoscopy. In a multicenter retrospective study of 249 cases, SEE‐AI provides visual cues that support physicians during interpretation while preserving physician‐led final decisions.

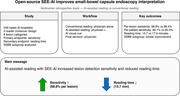

## 1 Introduction

Small bowel capsule endoscopy (CE) is a noninvasive modality that enables visualization of the entire small bowel using a swallowed capsule camera [1]. The PillCam SB3 capsule (Medtronic, Minneapolis, MN, USA) measures 11.4 mm × 26.2 mm, weighs 3 g, and acquires 2–6 frames per second depending on small bowel transit [2]. CE is widely used to evaluate small bowel diseases, including suspected small‐bowel bleeding (SSBB), inflammatory bowel disease, and neoplasia [3]. SSBB is the most common indication and frequently originates from small bowel lesions [4]. However, each PillCam SB3 study typically records approximately 8 h of video and around 50,000 images [5], imposing a substantial interpretive burden that may cause reader fatigue and missed lesions [6].

Applications of artificial intelligence (AI) in gastrointestinal endoscopy are expanding, including detection and characterization of neoplasia during upper endoscopy and colonoscopy [7, 8] and assessment of disease activity in ulcerative colitis [9]. Beyond diagnostic performance, AI may also reduce physician workload.

**FIGURE 1:**
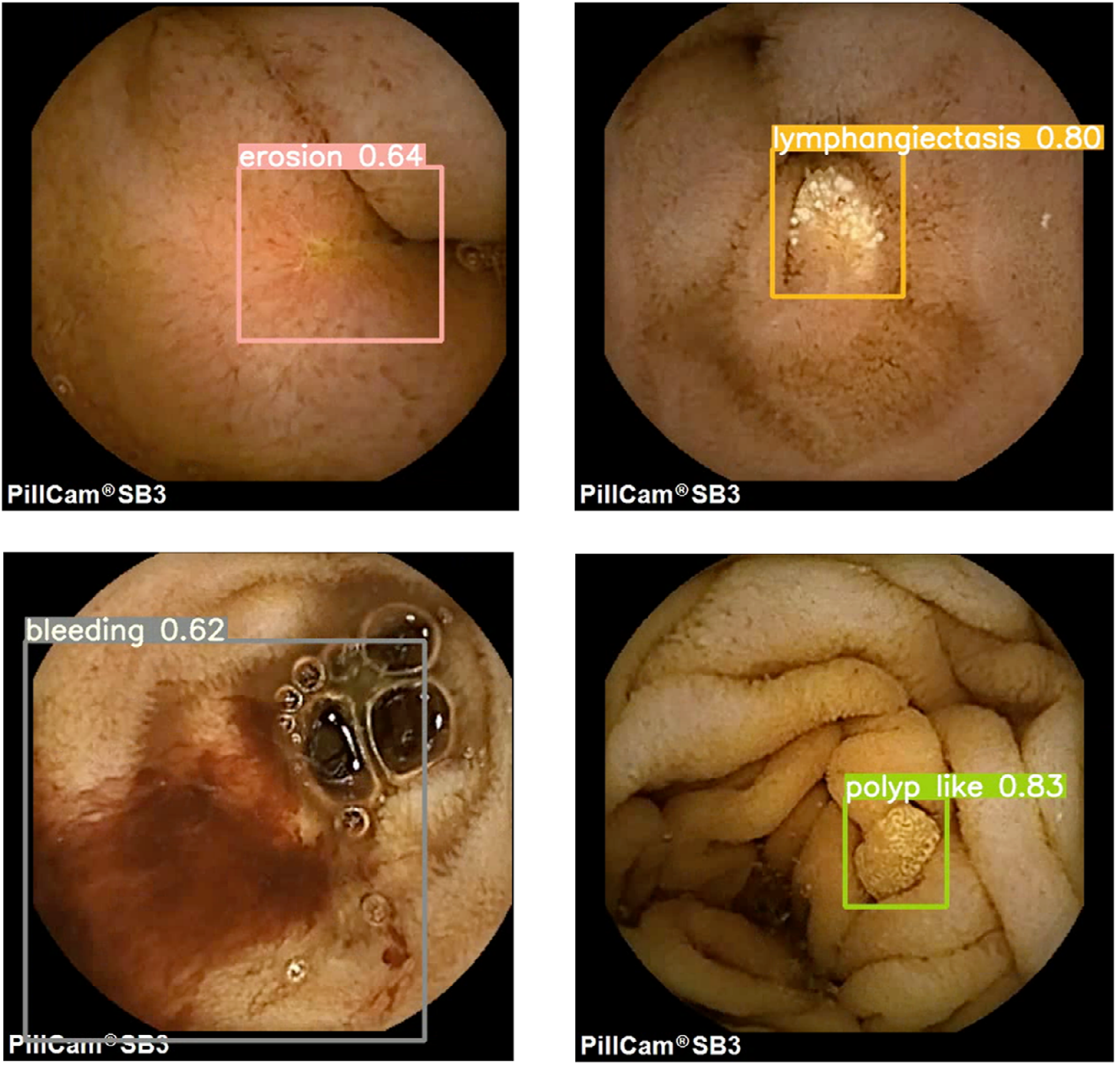
Representative capsule endoscopic images annotated by the SEE‐AI system. Each finding (erosion, lymphangiectasis, bleeding, and polyp‐like lesion) is labeled by a bounding box with an assigned confidence score.

AI is also promising for CE. The RAPID software for PillCam systems includes “TOP100,” which selects 100 frames most likely to contain potential lesions [10, 11]. The Navicam SB system (Ankon, Wuhan, China) includes “ProScan,” which automatically identifies and marks lesions [12]. Although previous studies have reported encouraging results, most were single‐center, used limited datasets, relied on nonpublic algorithms, or focused on specific lesion types. We previously developed SEE‐AI, an object detection model based on YOLOv5 that detects and classifies small bowel lesions [13, 14]. SEE‐AI is publicly available as an open‐source model [15] and overlays bounding boxes with confidence scores (Figure 1 and Video S1). While our previous work demonstrated lesion‐specific sensitivity, whether AI‐assisted reading improves overall diagnostic performance compared with conventional reading has not been evaluated.

Accordingly, using images collected from multiple institutions, we compared AI‐assisted and conventional reading, evaluating lesion detection sensitivity and reading time to clarify the feasibility of clinical implementation.

## 2 Methods

### 2.1 Study Design and Participants

We retrospectively analyzed 249 CE examinations performed with the PillCam SB3 system between January 2007 and September 2022 at six institutions: Fukuoka Red Cross Hospital, Hamanomachi Hospital, Kyushu Central Hospital of the Mutual Aid Association of Public School Teachers, Matsuyama Red Cross Hospital, Onga Nakama Medical Association Onga Hospital, and Yamaguchi Red Cross Hospital. The study was approved by the Institutional Review Board of Kyushu University (approval No. 22344‐00). Participation was solicited using an opt‐out approach, and anonymized CE video data and clinical information were retrospectively collected.

### 2.2 Video Processing and AI Inference

For each case, the segment from duodenal entry to cecal arrival was extracted using RAPID software for PillCam. Videos were exported in MPG format, in which each unique image is stored as five consecutive frames (i.e., four of every five frames are duplicates). To facilitate review and to avoid overcounting redundant frames, the MPG files were converted to MP4 by removing duplicate frames (downsampling). Conversion was performed on macOS 12 (Apple Inc., Cupertino, CA, USA) using Terminal 2.12 and FFmpeg v4.4.1 (FFmpeg Developers; https://ffmpeg.org) with the following command: ffmpeg ‐i input.mpg ‐r 5 output.mp4.

These MP4 files were used for conventional reading. The same videos were uploaded to Google Drive [16] and analyzed on Google Colaboratory (Google Colab), a cloud‐based Python environment with GPU acceleration [17]. SEE‐AI inference used a predefined confidence threshold of 0.1 to prioritize sensitivity, consistent with our previous validation study [14]. In that study, the relationship between confidence rate and model performance was evaluated, and the F1‐score increased rapidly at confidence rates ≤0.1 before showing a more gradual increase. Because SEE‐AI was intended to serve as a reading aid in which physicians review AI‐generated annotations, we selected this low threshold a priori to reduce missed lesions rather than to maximize F1‐score in the present dataset. Based on the AI output, we generated annotated videos with bounding boxes for AI‐assisted reading.

### 2.3 AI Model and Training

SEE‐AI is an open‐source model trained on anonymized small‐bowel CE videos from 954 patients who underwent PillCam SB3 examinations at Kyushu University Hospital between September 2014 and June 2021. The training dataset comprised 18,481 images across 41 confirmed disease categories, including 12,320 lesion images with 23,033 annotations and 6161 images of normal small‐bowel mucosa (Figure S1 and Table S1) [14].

### 2.4 Reading Protocol

**FIGURE 2:**
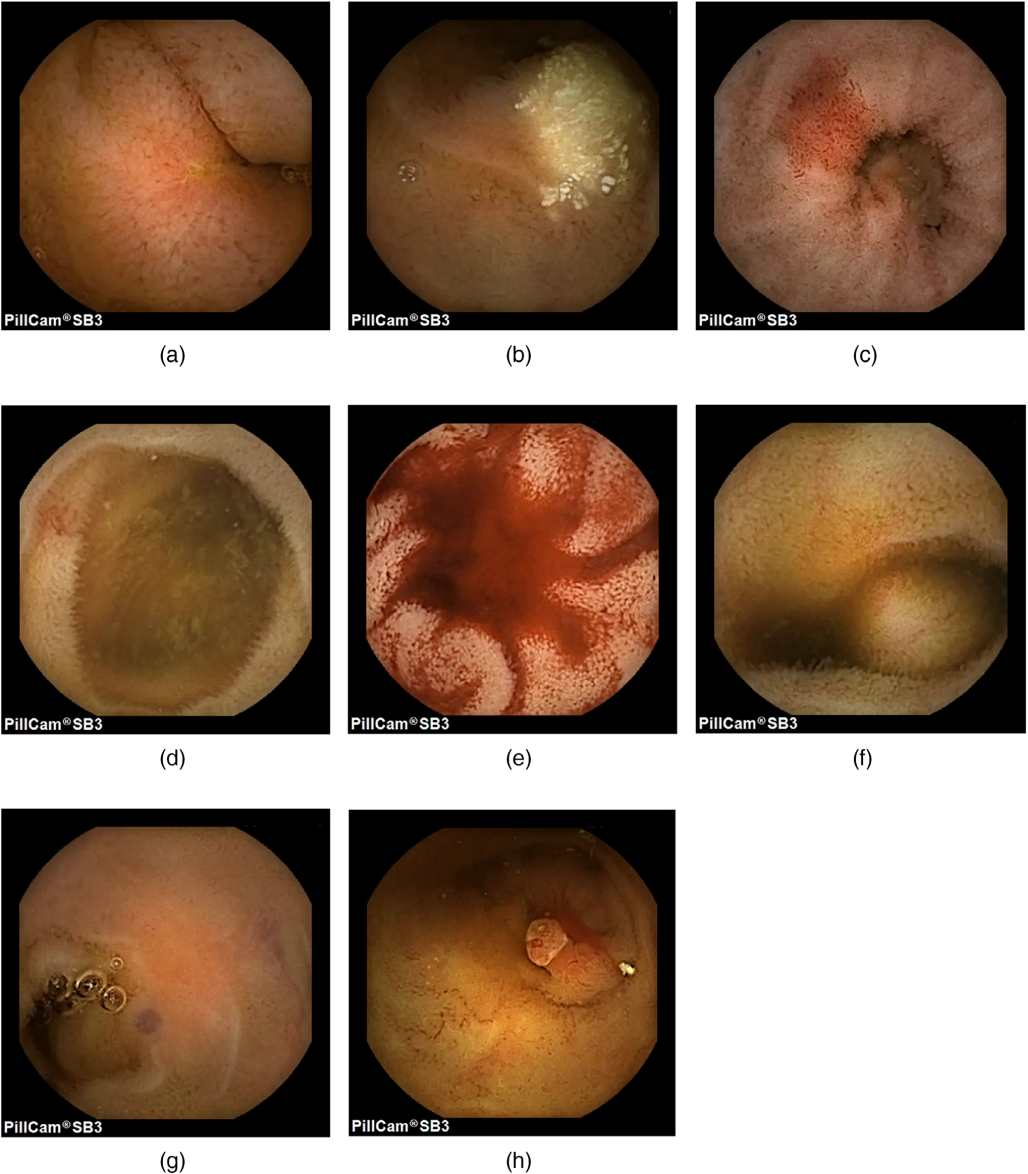
Representative images of eight types of lesions. (a) Erosion; (b) Lymphangiectasis; (c) Redness; (d) Angioectasia; (e) Bleeding; (f) Submucosal tumor; (g) Venous lesion; (h) Polyp‐like lesion.

The 249 cases were divided by case ID into two groups: Group A (IDs 1–125) and Group B (IDs 126–249). Two board‐certified endoscopists of the Japan Gastroenterological Endoscopy Society (Satoshi Miyazono and Takuto Saiki), each with more than 5 years of CE reading experience, participated in a crossover design: reader Satoshi Miyazono performed AI‐assisted reading for Group A and conventional reading for Group B, whereas reader Takuto Saiki performed conventional reading for Group A and AI‐assisted reading for Group B. Within each group, case order was randomized. To minimize potential learning effects, conventional and AI‐assisted reading were alternated so that the same reader did not perform the two methods consecutively. Readers were blinded to clinical information for all readings. Target lesions comprised eight categories: erosion, lymphangiectasis, redness, angioectasia, bleeding, submucosal tumor (SMT), venous lesion, and polyp‐like lesion (Figure 2; Table 1). Representative examples of redness and angioectasia are provided in Figure S2 to facilitate morphological distinction between these two lesion types. When a lesion was identified, readers recorded the finding and total reading time (minutes). For AI‐assisted reading, endoscopists reviewed the entire video with AI‐generated bounding‐box overlays and were not limited to flagged frames. In both methods, videos were manually reviewed without automatic playback.

**TABLE 1:** Definitions of the lesions.

Classification of lesions	Definition of lesions
Erosion	Areas of mucosal damage, such as erosions, ulcers, and notches
Lymphangiectasis	Areas containing lymphatic vessels larger than a point
Redness	A nonspecific erythematous color change without identifiable vascular structures or typical features of vascular dilatation
Angioectasia	A focal erythematous lesion with a visible cluster of dilated superficial vessels, including spider‐like or arborizing vascular patterns
Bleeding	Areas of apparent hemorrhage, exclude bile‐colored intestinal fluid
Submucosal tumor (SMT)	Areas resembling submucosal tumors
Venous lesion	Areas with venous structures
Polyp‐like lesion	Elevated lesions with a base or areas of suspected adenoma

Target lesions comprised eight categories: erosion, lymphangiectasis, redness, angioectasia, bleeding, submucosal tumor (SMT), venous lesion, and polyp‐like lesion.

### 2.5 Handling of Duplicate Lesions

When the same lesion appeared in multiple frames, identity was determined based on morphology, location, and temporal proximity. Lesions judged identical were counted once, and duplicates were excluded.

### 2.6 Reference Standard (Adjudication)

Findings recorded concordantly on AI‐assisted and conventional readings were considered “correct.” For discordant results, a third board‐certified endoscopist (Hisamitsu Kaku; >5 years of CE reading experience) adjudicated correctness.

### 2.7 Definition of False Positives

Diagnostic performance was assessed within an AI‐assisted workflow in which endoscopists retained final responsibility for interpretation. Reader‐level false positives were defined as findings recorded as lesions but not confirmed by the reference standard. AI‐generated boxes rejected by readers were not counted as reader‐level false positives. Separately, all AI‐generated bounding boxes were classified according to whether they corresponded to confirmed lesions; boxes without confirmed lesion correspondence were defined as algorithmic false‐positive boxes.

### 2.8 Outcomes and Statistical Analysis

The primary endpoints were lesion detection sensitivity on a per‐lesion and per‐patient basis. Secondary endpoints included per‐lesion and per‐patient specificity, positive predictive value, negative predictive value, overall accuracy, and reading time. Sensitivity was compared using McNemar's test, and reading time was compared using the Wilcoxon signed‐rank test. A two‐sided p < 0.05 was considered statistically significant.

Because SSBB is the most frequent indication for CE, we conducted a prespecified subgroup analysis limited to SSBB cases, comparing detection sensitivity and reading time for hemorrhagic lesions. Hemorrhagic lesions were defined according to the Saurin classification as P1 (uncertain hemorrhagic potential) and P2 (high hemorrhagic potential) [18] [Table S2]. The P1 + P2 group included erosion, redness, angioectasia, bleeding, and polyp‐like lesions. All analyses were performed using JMP Pro 17 (SAS Institute Inc., Cary, NC, USA).

## 3 Results

### 3.1 Patient Characteristics

Among the 249 cases, the median age was 67 years (interquartile range [IQR], 49–76); 145 patients were male (58.2%), and 104 were female (41.8%). The median number of frames in MP4 files was 10,071 (IQR, 6806–13,931). The indications for CE were SSBB in 131 cases (52.6%), inflammatory bowel disease in 43 (17.3%), and suspected neoplastic lesion in 11 (4.4%) (Table 2). Overall, 213 cases (85.5%) contained at least one of the eight target lesion types, whereas 36 (14.5%) had none. Across all readings, the following lesion counts were recorded (detected by AI‐assisted and/or conventional reading): erosions in 98 patients (825 lesions), lymphangiectasis in 105 (200 lesions), redness in 69 (187 lesions), angioectasia in 57 (92 lesions), bleeding in 31 (74 lesions), SMT in 43 (64 lesions), venous lesions in 38 (55 lesions), and polyp‐like lesions in 27 (53 lesions). The number of cases with each lesion type in Groups A and B is shown in Table S3.

**TABLE 2:** Baseline characteristics of patients (*n* = 249).

Characteristics	
Median age, years (IQR)	67 (49–76)
Male	145 (58.2%)
Median number of video frames in MP4 files (IQR)	10,071 (6806–13,931)
Cases with at least one of the eight types of lesions	213 (85.5%)
Indications for CE	
SSBB	131 (52.6%)
Inflammatory bowel disease	43 (17.3%)
─ Crohn's disease	38 (15.3%)
─ Behcet's disease	3 (1.2%)
─ Ulcerative colitis	1 (0.4%)
─ IBD‐U (unclassified)	1 (0.4%)
Neoplastic lesion	11 (4.4%)
─ Follicular lymphoma	4 (1.6%)
─ Metastatic tumor	2 (0.8%)
─ Diffuse large B‐cell lymphoma	1(0.4%)
─ Cronkhite–Canada syndrome	1 (0.4%)
─ Cowden syndrome	1 (0.4%)
─ Familial adenomatous polyposis	1 (0.4%)
─ Inflammatory fibroid polyp	1 (0.4%)
Other	61 (24.5%)
Unknown	3 (1.2%)

Age, sex, and indication for CE were collected. The number of video frames in the segment from duodenal entry to cecal arrival was measured. SSBB, suspected small‐bowel bleeding; IQR, interquartile range.

### 3.2 Lesion Detection Sensitivity (Overall Analysis)

Of the 1550 total lesions, 18 (1.16%) were not detected during AI‐assisted reading, whereas 211 (13.6%) were not detected during conventional reading. Per‐lesion sensitivity was 98.8% (1532/1550) for AI‐assisted reading versus 86.4% (1339/1550) for conventional reading (*p* < 0.0001). This higher sensitivity of AI‐assisted reading was observed across all eight lesion categories (Table 3). Per‐patient sensitivity was 99.1% (464/468) for AI‐assisted reading versus 80.3% (376/468) for conventional reading (*p* < 0.0001; Table 4). All lesions recorded during AI‐assisted reading had been flagged by SEE‐AI bounding boxes; no lesion was newly identified by the reader alone without an AI flag.

**TABLE 3:** Comparison of per‐lesion detection between artificial intelligence (AI)‐assisted and conventional readings in the overall cohort.

Classification of lesions		AI‐assisted reading					Conventional reading					*p*‐value^*^
	n	Sensitivity	Specificity	PPV	NPV	Accuracy	Sensitivity	Specificity	PPV	NPV	Accuracy	(Sensitivity)
Erosion	825	0.995	0.987	0.998	0.974	0.994	0.895	0.993	0.999	0.636	0.91	**<0.0001**
Lymphangiectasis	200	0.98	1	1	0.973	0.988	0.87	1	1	0.848	0.925	**<0.0001**
Redness	187	0.973	1	1	0.973	0.986	0.845	1	1	0.863	0.921	**<0.0001**
Angioectasia	92	0.967	1	1	0.985	0.989	0.609	1	1	0.842	0.873	**<0.0001**
Bleeding	74	0.986	1	1	0.995	0.997	0.892	1	1	0.965	0.973	**0.0196**
SMT	64	1	1	1	1	1	0.859	0.995	0.982	0.958	0.963	**0.0027**
Venous lesion	55	0.982	1	1	0.995	0.996	0.8	1	1	0.95	0.959	**0.0039**
Polyp‐like lesion	53	1	1	1	1	1	0.906	1	1	0.978	0.982	**0.0253**
Total	1550	0.988	0.999	0.999	0.988	0.994	0.864	0.999	0.999	0.879	0.931	**<0.0001**

NPV, Negative predictive value; PPV, Positive predictive value.

* McNemar test. Significant *p*‐values are indicated in bold.

**TABLE 4:** Comparison of per‐patient detection between artificial intelligence (AI)‐assisted and conventional readings in the overall cohort.

Classification of lesions		AI‐assisted reading					Conventional reading					*p*‐value^*^
	*n*	Sensitivity	Specificity	PPV	NPV	Accuracy	Sensitivity	Specificity	PPV	NPV	Accuracy	(Sensitivity)
Erosion	98	1	0.987	0.98	1	0.992	0.765	1	1	0.868	0.908	**<0.0001**
Lymphangiectasis	105	0.99	1	1	0.993	0.996	0.829	1	1	0.889	0.928	**<0.0001**
Redness	69	0.971	1	1	0.989	0.992	0.812	1	1	0.933	0.948	**0.0045**
Angioectasia	57	0.982	1	1	0.995	0.996	0.649	1	1	0.906	0.92	**<0.0001**
Bleeding	31	1	1	1	1	1	0.871	1	1	0.982	0.984	**0.0455**
SMT	43	1	1	1	1	1	0.907	1	1	0.981	0.984	**0.0455**
Venous lesion	38	1	1	1	1	1	0.842	1	1	0.972	0.976	**0.0143**
Polyp‐like lesion	27	1	1	1	1	1	0.852	1	1	0.982	0.984	**0.0455**
Total	468	0.991	0.999	0.996	0.997	0.997	0.803	1	1	0.943	0.954	**<0.0001**

NPV, Negative predictive value; PPV, Positive predictive value.

* McNemar test. Significant *p*‐values are indicated in bold.

### 3.3 AI‐generated Bounding Boxes and Algorithmic False Positives

Across the 249 analyzed videos, SEE‐AI generated a total of 365,959 bounding boxes, corresponding to a mean of 1469.7 boxes per case. Of these, 134,061 boxes corresponded to actual lesions confirmed by the reference standard, whereas 231,898 boxes did not correspond to confirmed lesions and were therefore classified as algorithmic false‐positive boxes. The lesion‐correspondence rate at the bounding‐box level was 36.6% (134,061/365,959), and the algorithmic false‐positive box rate was 63.4% (231,898/365,959).

### 3.4 Reading Time

**FIGURE 3:**
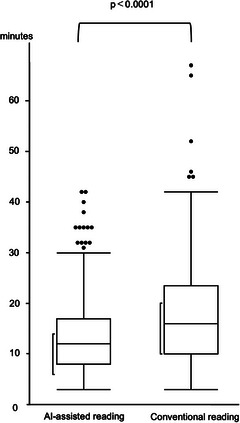
Comparison of reading time between artificial intelligence (AI)‐assisted and conventional readings in the overall analysis.

The mean reading time was 13.7 min (range, 3–42) for AI‐assisted reading and 17.9 min (range, 3–67) for conventional reading, which was significantly shorter with AI assistance (*p* < 0.0001; Figure 3).

### 3.5 Subgroup Analysis of SSBB

**FIGURE 4:**
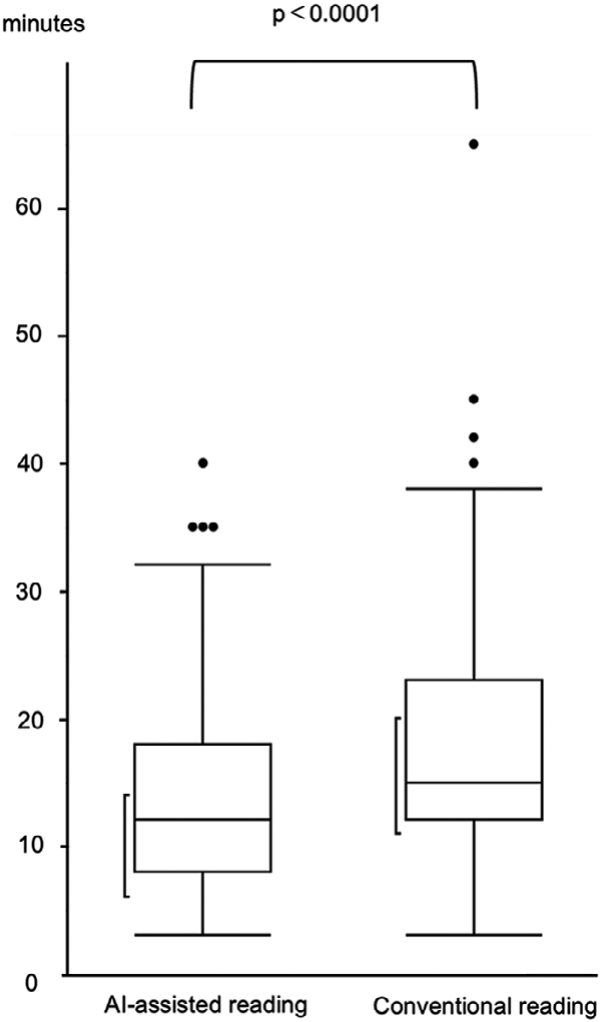
Comparison of reading time between artificial intelligence (AI)‐assisted and conventional readings in suspected small‐bowel bleeding (SSBB) cases.

In the 131 SSBB cases, per‐lesion sensitivity for Saurin P1 + P2 lesions was 98.2% (439/447) for AI‐assisted reading versus 82.8% (370/447) for conventional reading (*p* < 0.0001; Table 5). Per‐patient sensitivity was likewise higher with AI‐assisted reading: 98.6% (145/147) compared with 73.5% (108/147) for conventional reading (*p* < 0.0001; Table 6). Mean reading time was 14.1 min (range, 3–40) for AI‐assisted reading and 18.0 min (range, 3–65) for conventional reading (*p* < 0.0001; Figure 4).

**TABLE 5:** Comparison of per‐lesion detection between artificial intelligence (AI)‐assisted and conventional readings in suspected small‐bowel bleeding (SSBB) cases.

Classification of lesions		AI‐assisted reading					Conventional reading					*p*‐value^*^
	*n*	Sensitivity	Specificity	PPV	NPV	Accuracy	Sensitivity	Specificity	PPV	NPV	Accuracy	(Sensitivity)
Erosion	204	0.99	0.98	0.99	0.98	0.987	0.882	1	1	0.797	0.919	**<0.0001**
Redness	92	0.978	1	1	0.979	0.989	0.793	1	1	0.833	0.898	**0.0002**
Angioectasia	67	0.955	1	1	0.969	0.981	0.642	1	1	0.795	0.85	**<0.0001**
Bleeding	63	0.984	1	1	0.991	0.994	0.889	1	1	0.94	0.959	**0.0339**
Polyp‐like lesion	21	1	1	1	1	1	0.857	1	1	0.976	0.979	0.0833
Total (P1+P2)	447	0.982	0.996	0.995	0.985	0.99	0.828	1	1	0.869	0.920	**<0.0001**

NPV, Negative predictive value; PPV, Positive predictive value.

* McNemar test. Significant *p*‐values are indicated in bold.

**TABLE 6:** Comparison of per‐patient detection between artificial intelligence (AI)‐assisted and conventional readings in suspected small‐bowel bleeding (SSBB) cases.

Classification of lesions		AI‐assisted reading					Conventional reading					*p*‐value^*^
	*n*	Sensitivity	Specificity	PPV	NPV	Accuracy	Sensitivity	Specificity	PPV	NPV	Accuracy	(Sensitivity)
Erosion	38	1	0.978	0.95	1	0.985	0.658	1	1	0.877	0.901	**0.0003**
Redness	38	0.974	1	1	0.989	0.992	0.763	1	1	0.912	0.931	**0.0114**
Angioectasia	38	0.974	1	1	0.989	0.992	0.711	1	1	0.894	0.916	**0.0039**
Bleeding	22	1	1	1	1	1	0.864	1	1	0.973	0.977	0.0833
Polyp‐like lesion	11	1	1	1	1	1	0.727	1	1	0.976	0.977	0.0833
Total (P1+P2)	147	0.986	0.996	0.986	0.996	0.994	0.735	1	1	0.929	0.940	**<0.0001**

NPV, Negative predictive value; PPV, Positive predictive value.

* McNemar test. Significant *p*‐values are indicated in bold.

## 4 Discussion

In this multicenter study of 249 CE cases, we compared AI‐assisted reading using our open‐source SEE‐AI model with conventional reading. The cohort predominantly consisted of patients undergoing CE for SSBB, and a total of 1550 lesions across eight categories (erosion, lymphangiectasis, redness, angioectasia, bleeding, SMT, venous lesion, and polyp‐like lesion) were analyzed. AI‐assisted reading demonstrated higher sensitivity than conventional reading on both a per‐lesion basis (98.8% vs. 86.4%) and per‐patient basis (99.1% vs. 80.3%), with consistently improved detection across all lesion types. Furthermore, AI‐assisted reading significantly reduced reading time (13.7 vs. 17.9 min; *p* < 0.0001). In SSBB cases, AI‐assisted reading similarly improved sensitivity for Saurin P1 + P2 lesions (per‐lesion 98.2%, per‐patient 98.6%) while also shortening reading time. Collectively, SEE‐AI appears to enhance both diagnostic performance and efficiency in routine CE interpretation.

Lesion detection sensitivity was consistently higher with AI‐assisted reading in both per‐lesion and per‐patient analyses, in the overall cohort as well as in SSBB cases. Ding et al. reported per‐lesion and per‐patient sensitivities of 99.90% versus 76.89% and 99.88% versus 74.57%, respectively [19], whereas Xie et al. reported per‐lesion sensitivities of 79.3% versus 70.7% [20]. For bleeding‐related indications, Giordano et al. evaluated the TOP100 function of the PillCam SB3 and found that 88.35% of Saurin P2 lesions identified by conventional reading were detected [11]. Spada et al. compared the Navicam ProScan system with conventional reading in suspected small bowel bleeding and reported P1 + P2 lesion sensitivities of 73.7% versus 62.4% [21]. Together, these studies demonstrate that AI‐assisted reading improves lesion detection sensitivity, consistent with the present findings. During CE interpretation, decreased reader concentration due to prolonged review can lead to missed lesions even when visible on the video [6]. The marked improvement in sensitivity with AI‐assisted reading in our study supports the ability of SEE‐AI to reduce missed lesions. Given that SSBB is the principal indication for CE [4], demonstrating a benefit specifically in SSBB highlights the clinical relevance of SEE‐AI. Nevertheless, a small number of lesions were detected only by conventional reading, indicating that AI output should support—not replace—physician judgment.

Although AI‐assisted reading markedly improved sensitivity, a small number of lesions were still missed. In conventional reading, angioectasia showed relatively lower detection, likely due to its small and flat morphology. In contrast, AI assistance improved the detection of such subtle lesions. Lesions missed during AI‐assisted reading were often associated with bubbles or debris, peripheral localization, or partial visualization (Figure S3), which may hinder feature extraction and represent challenging scenarios for AI.

Reading time was significantly reduced overall and in the SSBB subgroup. Ding et al. analyzed 3,280 cases and reported mean reading times of 5.9 ± 2.23 min for AI‐assisted reading versus 96.6 ± 22.53 min for conventional reading [19]. Xie et al. reported similar results (5.4 ± 1.5 vs. 51.4 ± 11.6 min) [20]. In overt bleeding cases, Giordano et al. found reading times of 1.9 versus 23 min with the PillCam SB3 TOP100 function [11], whereas Spada et al. reported 3.8 ± 3.3 versus 33.7 ± 22.9 min using Navicam ProScan [21]. In our study, conventional reading was faster than in most prior reports (17.9 min overall; 18.0 min in SSBB). Although typical reading times for small bowel CE have been reported as 30–90 min [22], Omori et al. demonstrated that reading time depends on both reader experience and capsule generation: beginners required 40.2 ± 10.1 versus 23.7 ± 6.7 min for SB2 versus SB3, whereas experts required 23.2 ± 5.6 versus 11.1 ± 2.9 min [23]. Our readers were experienced endoscopists, and all examinations used SB3, likely contributing to shorter conventional reading times. Conversely, our AI‐assisted reading times were longer than those reported in some previous AI studies, which often limited review to AI‐flagged segments. Because AI‐based lesion detection still has a non‐zero miss rate, clinicians must continue reviewing non‐flagged segments in real‐world practice. In this study, although the entire video was reviewed even during AI‐assisted reading, the bounding‐box overlays provided by the AI served as visual cues that enabled readers to focus their attention more efficiently on lesion‐suspected areas. This ability to prioritize the evaluation of suspicious regions is considered to be a primary factor contributing to the reduction in reading time. Under these pragmatic conditions, AI‐assisted reading significantly reduced reading time, supporting the potential of SEE‐AI to reduce workload in daily clinical practice.

The burden of AI‐generated candidate findings should be considered when interpreting the reduction in reading time. Although SEE‐AI generated many algorithmic false‐positive boxes, AI‐assisted reading still significantly shortened reading time compared with conventional reading. Experienced readers may have rapidly dismissed non‐lesion boxes by considering morphology, temporal continuity, and surrounding frames, while using lesion‐associated boxes as visual cues. However, excessive AI outputs may increase cognitive burden, particularly for less experienced readers. Further model refinement and confidence‐threshold optimization are needed to balance sensitivity with interpretive workload.

A key strength of this study is the use of SEE‐AI, a publicly released, pretrained CE AI model validated using multicenter clinical data [14, 15]. Although many CE AI models have been reported, few are available for independent use or external validation. To our knowledge, this is the first multicenter clinical evaluation of an open‐source, pretrained CE AI model, supporting transparency and reproducibility.

This study has several limitations. First, primary readings were performed by only two Japan Gastroenterological Endoscopy Society–certified endoscopists; therefore, inter‐reader variability—particularly regarding experience level—was not fully assessed. Prior studies have shown differences in detection rates between trainees and experts for erosions and ulcers [24]; future SEE‐AI studies should stratify findings by reader experience. Second, the high number of algorithmic false‐positive boxes remains an important limitation, particularly for less experienced readers. Further studies should evaluate how this burden affects real‐world interpretation efficiency across different levels of reader experience. Third, differentiating redness from angioectasia based solely on capsule findings may be imperfect, despite our definitions requiring visible dilated superficial vessels for angioectasia. Because balloon endoscopic confirmation was not performed, some misclassification cannot be excluded. Fourth, SEE‐AI inference currently requires GPU resources and approximately one hour for video extraction and annotated video generation, creating cost and operational burdens. Fifth, all cases were acquired with PillCam SB3; because image color tone and luminance differ between systems [25], cross‐platform validation is needed.

Future studies should evaluate readers with different experience levels, other capsule platforms, streamlined clinical workflows, and larger datasets with high‐quality annotations.

## 5 Conclusions

In CE, SEE‐AI improved both diagnostic performance and reading efficiency. AI‐assisted reading has the potential to serve as a new standard of care for the evaluation of small bowel disease.

## Author Contributions

**Satoshi Miyazono**: Conceptualization; Methodology; Data curation; Investigation; Validation; Formal analysis; Visualization; Project administration; Resources; Writing – original draft; Writing – review & editing. **Junji Umeno**: Conceptualization; Methodology; Data curation; Validation; Formal analysis; Supervision; Writing – review & editing. **Tomohiro Nagasue**: Conceptualization; Methodology; Data curation; Validation; Formal analysis; Supervision; Writing – review & editing. **Takuto Saiki**: Data curation; Investigation; Writing – review & editing. **Hisamitsu Kaku**: Data curation; Investigation; Writing – review & editing. **Takehiro Torisu**: Conceptualization; Methodology; Data curation; Validation; Formal analysis; Writing – review & editing. **Akihito Yokote**: Software; Data curation; Writing – review & editing. **Keisuke Kawasaki**: Data curation; Writing – review & editing. **Yutaro Ihara**: Data curation; Writing – review & editing. **Yuichi Matsuno**: Data curation; Writing – review & editing. **Noriyuki Imazu**: Data curation; Writing – review & editing. **Tomohiko Moriyama**: Data curation; Writing – review & editing. **Ahmed Nashaat Mohamed**: Data curation; Writing – review & editing. **Katsuya Hirakawa**: Data curation; Resources; Writing – review & editing. **Hajime Yamagata**: Data curation; Resources; Writing – review & editing. **Yasuharu Okamoto**: Data curation; Resources; Writing – review & editing. **Koichi Kurahara**: Data curation; Resources; Writing – review & editing. **Shinichiro Yada**: Data curation; Resources; Writing – review & editing. **Akira Harada**: Data curation; Resources; Writing – review & editing. **Tetsuro Ago**: Conceptualization; Methodology; Data curation; Validation; Formal analysis; Supervision; Writing – review & editing. All authors reviewed and approved the final manuscript.

## Conflicts of Interest

The authors declare no conflicts of interest.

## Funding

The authors have nothing to report.

## Ethics Statement

This retrospective study was approved by the Institutional Review Board of Kyushu University (approval No. 22344‐00). Participation was solicited using an opt‐out approach, and anonymized capsule endoscopy video data and clinical information were retrospectively collected.

## Consent

Obtained via an opt‐out method.

## Supporting information




**Supporting Video 1**: Sample video demonstrating the detection of erosions.

**Supporting Table 1**: Disease background of the dataset.
**Supporting Table 2**: Saurin classification: Categorization of small‐bowel lesions into three groups (P0, P1, and P2) according to their bleeding potential.
**Supporting Table 3**: Distribution of lesion types in Groups A and B.
**Supporting Figure 1**: Construction of the SEE‐AI training dataset.
**Supporting Figure 2**: Representative images of angioectasia and redness. Panels (a–c) show representative examples of angioectasia, whereas panels (d–f) show representative examples of redness.
**Supporting Figure 3**: Representative examples of lesions missed during AI‐assisted reading. Panels (a–d) show representative examples of lesions missed during AI‐assisted reading. (a) Ulcer with incomplete visualization and surrounding bubbles. (b) Lymphangiectasis with adjacent debris and bubbles. (c) Bleeding with surrounding bubbles and an overall darkened appearance. (d) Venous lesion partially visualized within the frame.
