# Supplementary material for: Improved Efficiency and Lesion Detection in Small Bowel Capsule Endoscopy Using the Open‐Source Artificial Intelligence Model SEE‐AI
Source: DEN Open. 2026 May 15;7:e70346. doi: 10.1002/deo2.70346 (PMC13177839; doi:10.1002/deo2.70346)
Supplement: Supplementary file 2 — Supporting Table 1: Disease background of the dataset. Supporting Table 2: Saurin classification: Categorization of small‐bowel lesions into three groups (P0, P1, and P2) according to their bleeding potential. Supporting Table 3: Distribution of lesion types in Groups A and B. Supporting Figure 1: Construction of the SEE‐AI training dataset. Supporting Figure 2: Representative images of angioectasia and redness. Panels (a–c) show representative examples of angioectasia, whereas panels (d–f) show representative examples of redness. Supporting Figure 3: Representative examples of lesions missed during AI‐assisted reading. Panels (a–d) show representative examples of lesions missed during AI‐assisted reading. (a) Ulcer with incomplete visualization and surrounding bubbles. (b) Lymphangiectasis with adjacent debris and bubbles. (c) Bleeding with surrounding bubbles and an overall darkened appearance. (d) Venous lesion partially visualized within the frame. [file DEO2-7-e70346-s002.zip › deo270346-sup-0002-SuppMat/deo270346-sup-0001-tableS1-S3.docx]

TableS1. Disease background of the dataset

| **Characteristics** | | |
| --- | --- | --- |
| Number of images | | 18,481 |
|  | With disease lesions | 12,320 |
|  | Number of annotations | 23,033 |
| **Cause** | |  |
|  | Follicular lymphoma | 77 |
|  | Crohn’s disease | 58 |
|  | Angioectasia | 39 |
|  | MALT lymphoma | 22 |
|  | Familial adenomatous polyposis | 23 |
|  | Diffuse large B-cell lymphoma | 17 |
|  | Cowden syndrome | 17 |
|  | Peutz-Jeghers syndrome | 14 |
|  | NSAIDs ulcer | 9 |
|  | GIST, Adenoma | 7 |
|  | Amyloidosis | 6 |
|  | IgA vasculitis, Anastomotic ulcer | 3 |
|  | Small intestine cancer, Bechet's disease, Intestinal tuberculosis, Protein-losing gastroenteropathy, Metastatic cancer, Arteriovenous malformation, Adult T-cell leukemia-lymphoma, Eosinophil enteritis, Chronic enteropathy associated with *SLCO2A1* gene | 2 |
|  | Radioactive enteritis, Ischemic enteritis, T cell lymphoma, Plasma cell tumor, Cryoglobulinemia, Intestinal emphysema, Polyarteritis nodosa, Scleroderma, Cavernous hemangioma, Olmesartan Associated Enteropathy, Cancer of unknown primary, Celiac disease, Sarcoma, Giardia, Ulcerative Colitis, Systemic lupus erythematosus, Neuroendocrine neoplasm, Cytomegalovirus enteritis | 1 |
|  | Unknown disease name | 196 |
| Disease backgrounds of collected images in the dataset. MALT; mucosa associated lymphoid tissue, NSAIDs; non-steroidal anti-inflammatory drug, GIST; gastrointestinal stromal tumor | | |

TableS2. Saurin classification

| Classification of lesions | Hemorrhagic potential | Examples of lesions |
| --- | --- | --- |
| P0 | No potential for bleeding | Submucosal veins, diverticula without visible blood, or nodules without mucosal break |
| P1 | Uncertain hemorrhagic potential | Red spots on the intestinal mucosa or small isolated erosions |
| P2 | High potential for bleeding | Angioectasia, large ulcerations, tumors, or varices, blood |

Hemorrhagic lesions were defined as P1 + P2 lesions, including erosion, redness, angioectasia

TableS3. Number of cases with each lesion type in Groups A and B

| Group | Angioectasia | Bleeding | Erosion | Lymphangiectasis | Polyp-like lesion | Redness | Submucosal tumor | Venous  lesion | Total |
| --- | --- | --- | --- | --- | --- | --- | --- | --- | --- |
| Group A  (n=125) | 29 (11.2%) | 17 (6.6%) | 55 (21.2%) | 62 (23.9%) | 17 (6.6%) | 33 (12.7%) | 28 (10.8%) | 18 (7.0%) | 259 |
| Group B  (n=124) | 28 (13.4%) | 14 (6.7%) | 43 (20.6%) | 43 (20.6%) | 10 (4.8%) | 36 (17.2%) | 15 (7.2%) | 20 (9.6%) | 209 |

Data are presented as the number of cases with each lesion type (n, % within each group).
