# Supplementary figures and images for: Improved Efficiency and Lesion Detection in Small Bowel Capsule Endoscopy Using the Open‐Source Artificial Intelligence Model SEE‐AI
Source: DEN Open. 2026 May 15;7:e70346. doi: 10.1002/deo2.70346 (PMC13177839; doi:10.1002/deo2.70346)

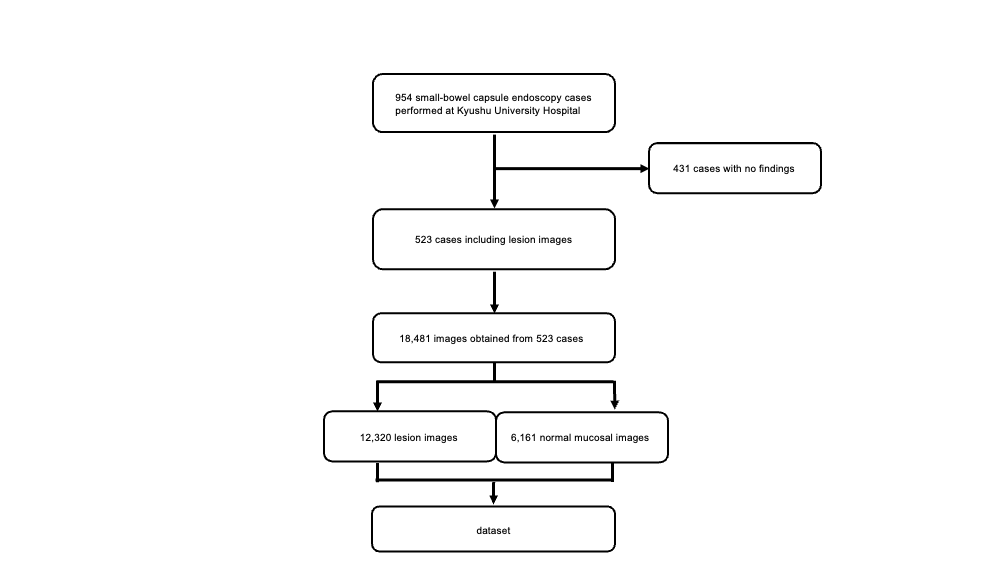

Supplement: Supplementary file 2 — Supporting Table 1: Disease background of the dataset. Supporting Table 2: Saurin classification: Categorization of small‐bowel lesions into three groups (P0, P1, and P2) according to their bleeding potential. Supporting Table 3: Distribution of lesion types in Groups A and B. Supporting Figure 1: Construction of the SEE‐AI training dataset. Supporting Figure 2: Representative images of angioectasia and redness. Panels (a–c) show representative examples of angioectasia, whereas panels (d–f) show representative examples of redness. Supporting Figure 3: Representative examples of lesions missed during AI‐assisted reading. Panels (a–d) show representative examples of lesions missed during AI‐assisted reading. (a) Ulcer with incomplete visualization and surrounding bubbles. (b) Lymphangiectasis with adjacent debris and bubbles. (c) Bleeding with surrounding bubbles and an overall darkened appearance. (d) Venous lesion partially visualized within the frame. [file DEO2-7-e70346-s002.zip › deo270346-sup-0002-SuppMat/deo270346-sup-0002-figureS1.tiff]

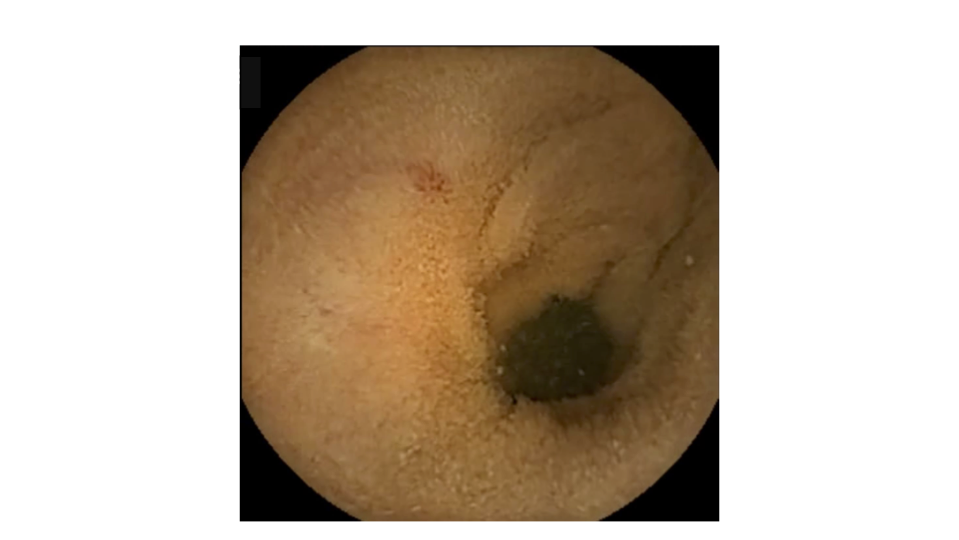

Supplement: Supplementary file 2 — Supporting Table 1: Disease background of the dataset. Supporting Table 2: Saurin classification: Categorization of small‐bowel lesions into three groups (P0, P1, and P2) according to their bleeding potential. Supporting Table 3: Distribution of lesion types in Groups A and B. Supporting Figure 1: Construction of the SEE‐AI training dataset. Supporting Figure 2: Representative images of angioectasia and redness. Panels (a–c) show representative examples of angioectasia, whereas panels (d–f) show representative examples of redness. Supporting Figure 3: Representative examples of lesions missed during AI‐assisted reading. Panels (a–d) show representative examples of lesions missed during AI‐assisted reading. (a) Ulcer with incomplete visualization and surrounding bubbles. (b) Lymphangiectasis with adjacent debris and bubbles. (c) Bleeding with surrounding bubbles and an overall darkened appearance. (d) Venous lesion partially visualized within the frame. [file DEO2-7-e70346-s002.zip › deo270346-sup-0002-SuppMat/deo270346-sup-0003-figureS2a.tiff]

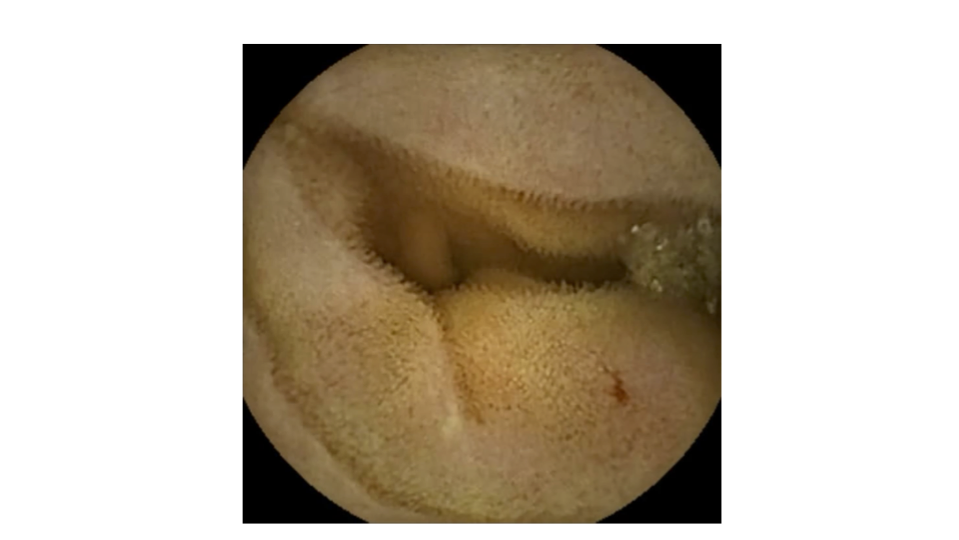

Supplement: Supplementary file 2 — Supporting Table 1: Disease background of the dataset. Supporting Table 2: Saurin classification: Categorization of small‐bowel lesions into three groups (P0, P1, and P2) according to their bleeding potential. Supporting Table 3: Distribution of lesion types in Groups A and B. Supporting Figure 1: Construction of the SEE‐AI training dataset. Supporting Figure 2: Representative images of angioectasia and redness. Panels (a–c) show representative examples of angioectasia, whereas panels (d–f) show representative examples of redness. Supporting Figure 3: Representative examples of lesions missed during AI‐assisted reading. Panels (a–d) show representative examples of lesions missed during AI‐assisted reading. (a) Ulcer with incomplete visualization and surrounding bubbles. (b) Lymphangiectasis with adjacent debris and bubbles. (c) Bleeding with surrounding bubbles and an overall darkened appearance. (d) Venous lesion partially visualized within the frame. [file DEO2-7-e70346-s002.zip › deo270346-sup-0002-SuppMat/deo270346-sup-0004-figureS2b.tiff]

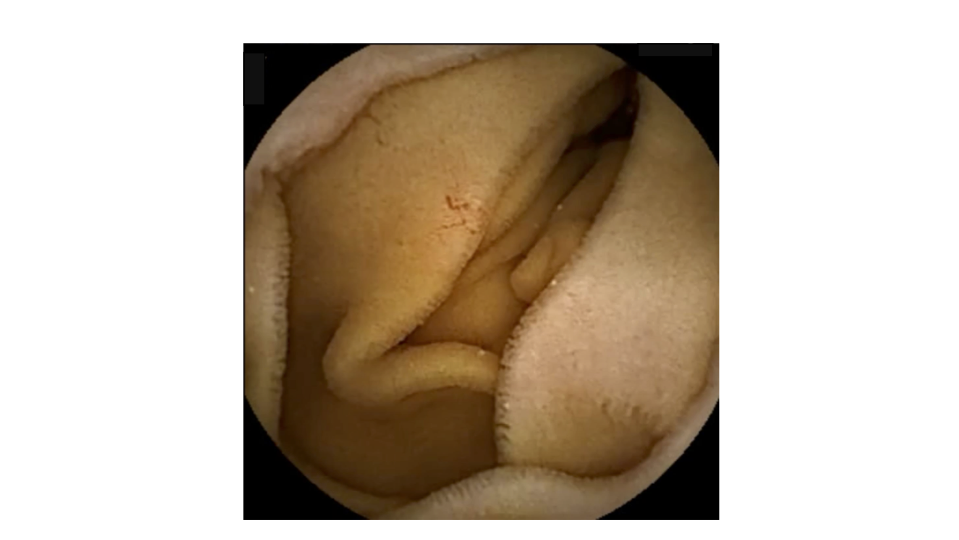

Supplement: Supplementary file 2 — Supporting Table 1: Disease background of the dataset. Supporting Table 2: Saurin classification: Categorization of small‐bowel lesions into three groups (P0, P1, and P2) according to their bleeding potential. Supporting Table 3: Distribution of lesion types in Groups A and B. Supporting Figure 1: Construction of the SEE‐AI training dataset. Supporting Figure 2: Representative images of angioectasia and redness. Panels (a–c) show representative examples of angioectasia, whereas panels (d–f) show representative examples of redness. Supporting Figure 3: Representative examples of lesions missed during AI‐assisted reading. Panels (a–d) show representative examples of lesions missed during AI‐assisted reading. (a) Ulcer with incomplete visualization and surrounding bubbles. (b) Lymphangiectasis with adjacent debris and bubbles. (c) Bleeding with surrounding bubbles and an overall darkened appearance. (d) Venous lesion partially visualized within the frame. [file DEO2-7-e70346-s002.zip › deo270346-sup-0002-SuppMat/deo270346-sup-0005-figureS2c.tiff]

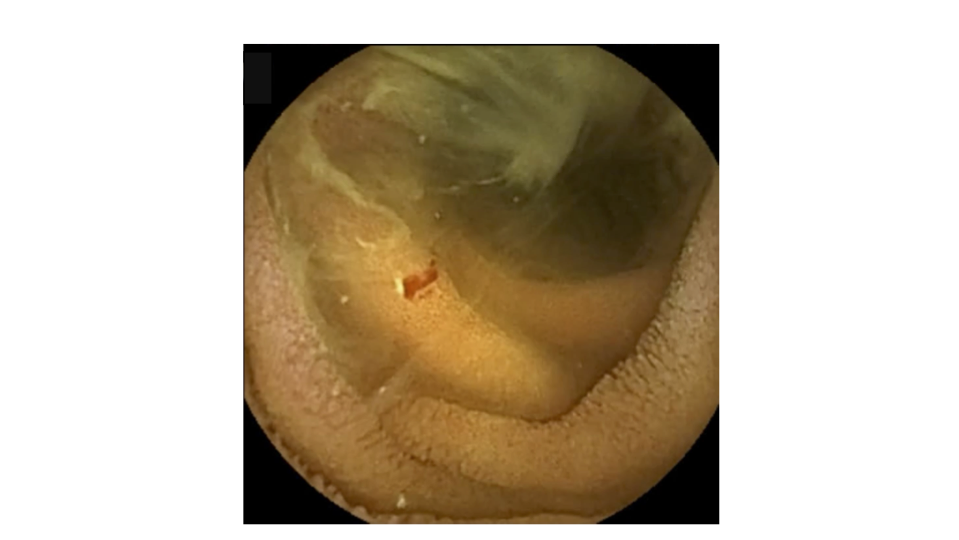

Supplement: Supplementary file 2 — Supporting Table 1: Disease background of the dataset. Supporting Table 2: Saurin classification: Categorization of small‐bowel lesions into three groups (P0, P1, and P2) according to their bleeding potential. Supporting Table 3: Distribution of lesion types in Groups A and B. Supporting Figure 1: Construction of the SEE‐AI training dataset. Supporting Figure 2: Representative images of angioectasia and redness. Panels (a–c) show representative examples of angioectasia, whereas panels (d–f) show representative examples of redness. Supporting Figure 3: Representative examples of lesions missed during AI‐assisted reading. Panels (a–d) show representative examples of lesions missed during AI‐assisted reading. (a) Ulcer with incomplete visualization and surrounding bubbles. (b) Lymphangiectasis with adjacent debris and bubbles. (c) Bleeding with surrounding bubbles and an overall darkened appearance. (d) Venous lesion partially visualized within the frame. [file DEO2-7-e70346-s002.zip › deo270346-sup-0002-SuppMat/deo270346-sup-0006-figureS2d.tiff]

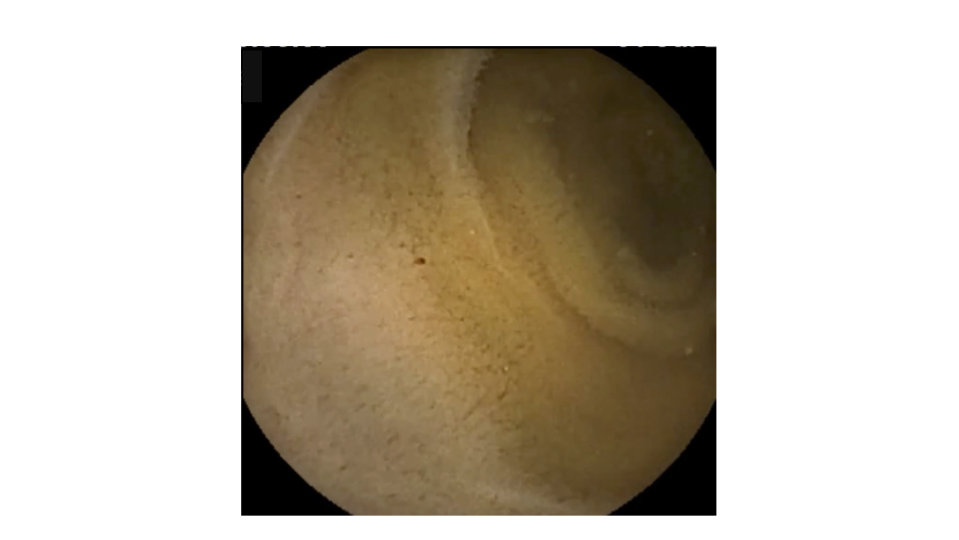

Supplement: Supplementary file 2 — Supporting Table 1: Disease background of the dataset. Supporting Table 2: Saurin classification: Categorization of small‐bowel lesions into three groups (P0, P1, and P2) according to their bleeding potential. Supporting Table 3: Distribution of lesion types in Groups A and B. Supporting Figure 1: Construction of the SEE‐AI training dataset. Supporting Figure 2: Representative images of angioectasia and redness. Panels (a–c) show representative examples of angioectasia, whereas panels (d–f) show representative examples of redness. Supporting Figure 3: Representative examples of lesions missed during AI‐assisted reading. Panels (a–d) show representative examples of lesions missed during AI‐assisted reading. (a) Ulcer with incomplete visualization and surrounding bubbles. (b) Lymphangiectasis with adjacent debris and bubbles. (c) Bleeding with surrounding bubbles and an overall darkened appearance. (d) Venous lesion partially visualized within the frame. [file DEO2-7-e70346-s002.zip › deo270346-sup-0002-SuppMat/deo270346-sup-0007-figureS2e.tiff]

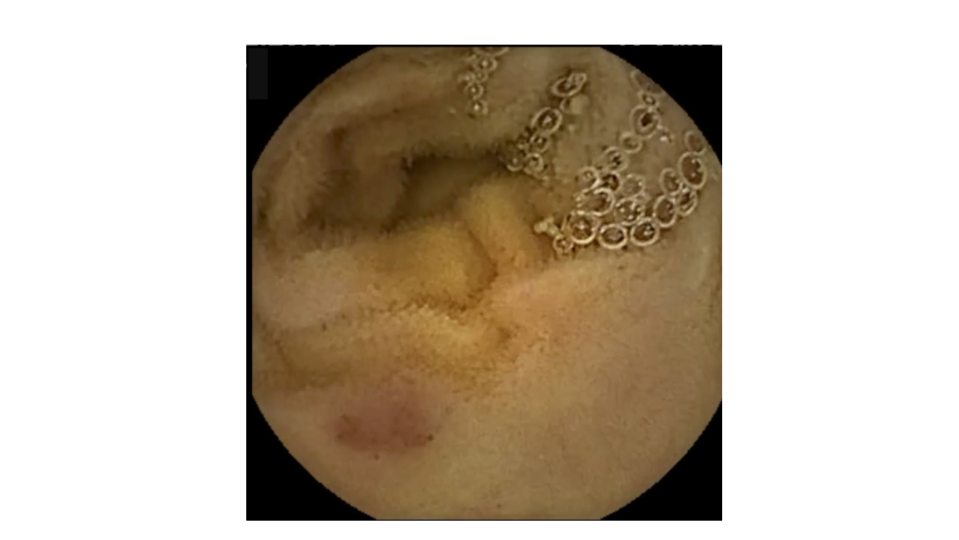

Supplement: Supplementary file 2 — Supporting Table 1: Disease background of the dataset. Supporting Table 2: Saurin classification: Categorization of small‐bowel lesions into three groups (P0, P1, and P2) according to their bleeding potential. Supporting Table 3: Distribution of lesion types in Groups A and B. Supporting Figure 1: Construction of the SEE‐AI training dataset. Supporting Figure 2: Representative images of angioectasia and redness. Panels (a–c) show representative examples of angioectasia, whereas panels (d–f) show representative examples of redness. Supporting Figure 3: Representative examples of lesions missed during AI‐assisted reading. Panels (a–d) show representative examples of lesions missed during AI‐assisted reading. (a) Ulcer with incomplete visualization and surrounding bubbles. (b) Lymphangiectasis with adjacent debris and bubbles. (c) Bleeding with surrounding bubbles and an overall darkened appearance. (d) Venous lesion partially visualized within the frame. [file DEO2-7-e70346-s002.zip › deo270346-sup-0002-SuppMat/deo270346-sup-0008-figureS2f.tiff]

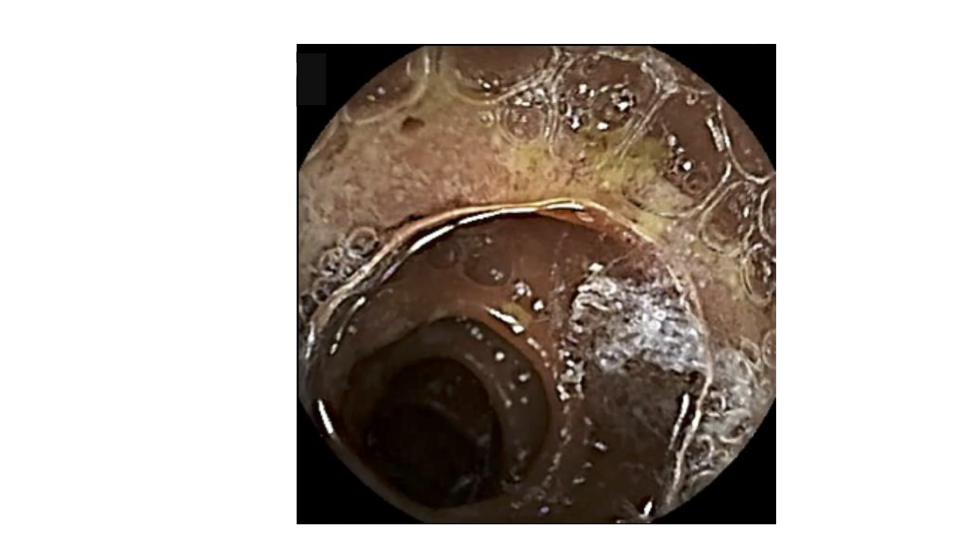

Supplement: Supplementary file 2 — Supporting Table 1: Disease background of the dataset. Supporting Table 2: Saurin classification: Categorization of small‐bowel lesions into three groups (P0, P1, and P2) according to their bleeding potential. Supporting Table 3: Distribution of lesion types in Groups A and B. Supporting Figure 1: Construction of the SEE‐AI training dataset. Supporting Figure 2: Representative images of angioectasia and redness. Panels (a–c) show representative examples of angioectasia, whereas panels (d–f) show representative examples of redness. Supporting Figure 3: Representative examples of lesions missed during AI‐assisted reading. Panels (a–d) show representative examples of lesions missed during AI‐assisted reading. (a) Ulcer with incomplete visualization and surrounding bubbles. (b) Lymphangiectasis with adjacent debris and bubbles. (c) Bleeding with surrounding bubbles and an overall darkened appearance. (d) Venous lesion partially visualized within the frame. [file DEO2-7-e70346-s002.zip › deo270346-sup-0002-SuppMat/deo270346-sup-0009-figureS3a.tiff]

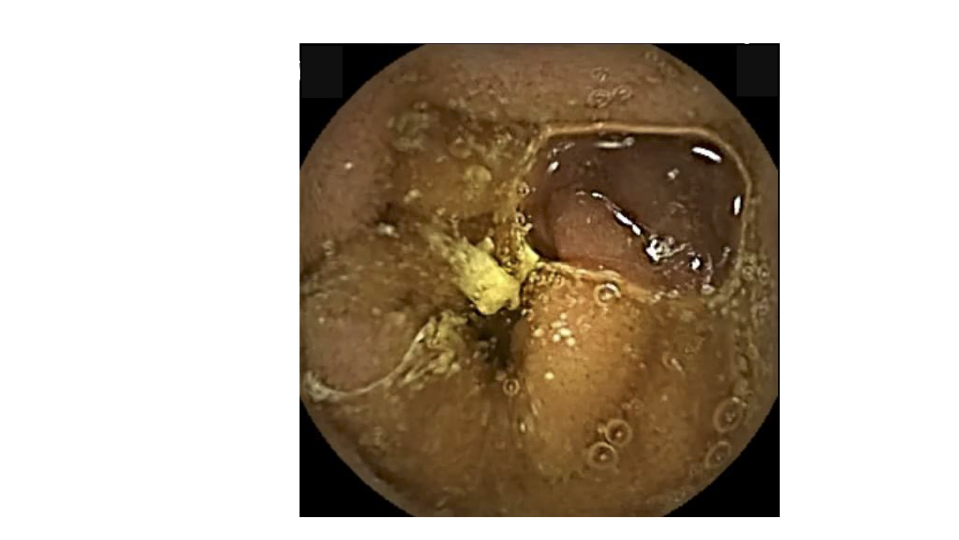

Supplement: Supplementary file 2 — Supporting Table 1: Disease background of the dataset. Supporting Table 2: Saurin classification: Categorization of small‐bowel lesions into three groups (P0, P1, and P2) according to their bleeding potential. Supporting Table 3: Distribution of lesion types in Groups A and B. Supporting Figure 1: Construction of the SEE‐AI training dataset. Supporting Figure 2: Representative images of angioectasia and redness. Panels (a–c) show representative examples of angioectasia, whereas panels (d–f) show representative examples of redness. Supporting Figure 3: Representative examples of lesions missed during AI‐assisted reading. Panels (a–d) show representative examples of lesions missed during AI‐assisted reading. (a) Ulcer with incomplete visualization and surrounding bubbles. (b) Lymphangiectasis with adjacent debris and bubbles. (c) Bleeding with surrounding bubbles and an overall darkened appearance. (d) Venous lesion partially visualized within the frame. [file DEO2-7-e70346-s002.zip › deo270346-sup-0002-SuppMat/deo270346-sup-0010-figureS3b.tiff]

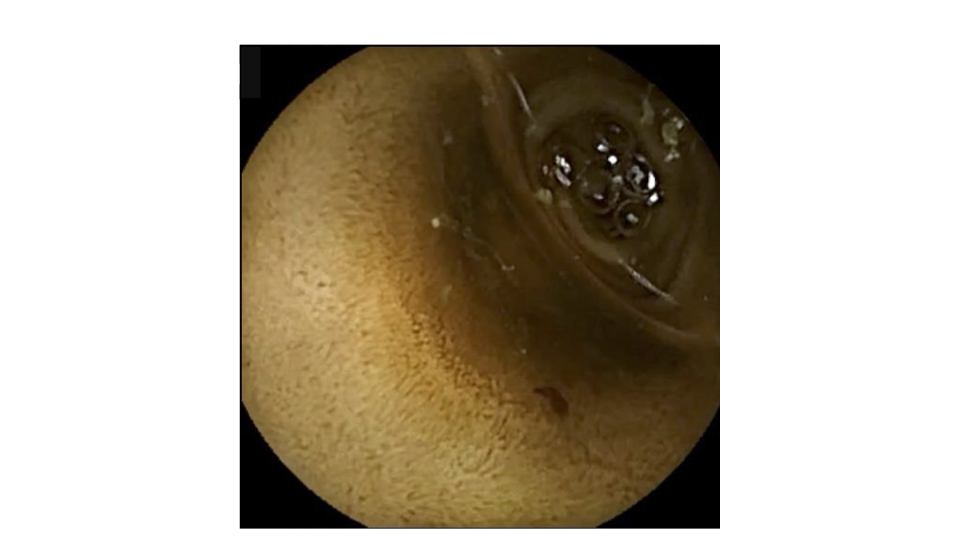

Supplement: Supplementary file 2 — Supporting Table 1: Disease background of the dataset. Supporting Table 2: Saurin classification: Categorization of small‐bowel lesions into three groups (P0, P1, and P2) according to their bleeding potential. Supporting Table 3: Distribution of lesion types in Groups A and B. Supporting Figure 1: Construction of the SEE‐AI training dataset. Supporting Figure 2: Representative images of angioectasia and redness. Panels (a–c) show representative examples of angioectasia, whereas panels (d–f) show representative examples of redness. Supporting Figure 3: Representative examples of lesions missed during AI‐assisted reading. Panels (a–d) show representative examples of lesions missed during AI‐assisted reading. (a) Ulcer with incomplete visualization and surrounding bubbles. (b) Lymphangiectasis with adjacent debris and bubbles. (c) Bleeding with surrounding bubbles and an overall darkened appearance. (d) Venous lesion partially visualized within the frame. [file DEO2-7-e70346-s002.zip › deo270346-sup-0002-SuppMat/deo270346-sup-0011-figureS3c.tiff]

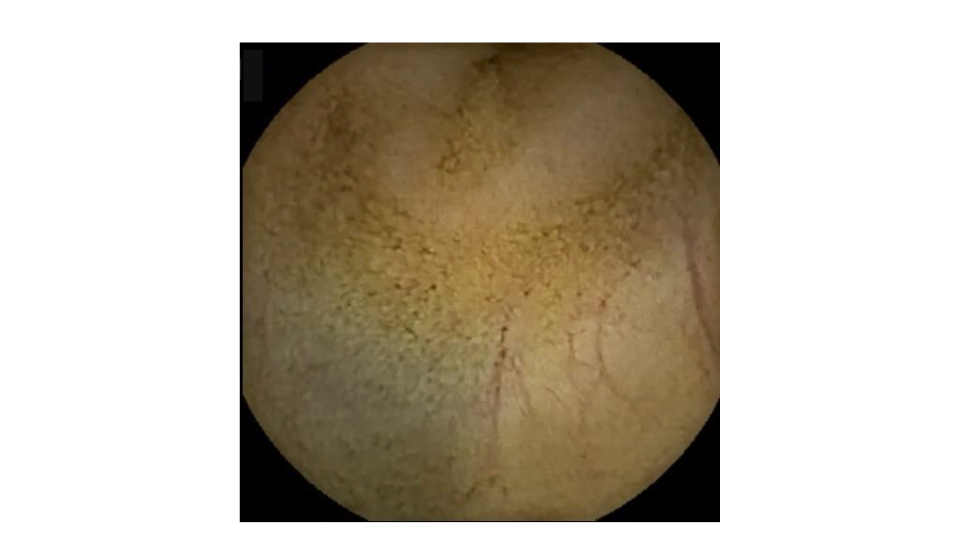

Supplement: Supplementary file 2 — Supporting Table 1: Disease background of the dataset. Supporting Table 2: Saurin classification: Categorization of small‐bowel lesions into three groups (P0, P1, and P2) according to their bleeding potential. Supporting Table 3: Distribution of lesion types in Groups A and B. Supporting Figure 1: Construction of the SEE‐AI training dataset. Supporting Figure 2: Representative images of angioectasia and redness. Panels (a–c) show representative examples of angioectasia, whereas panels (d–f) show representative examples of redness. Supporting Figure 3: Representative examples of lesions missed during AI‐assisted reading. Panels (a–d) show representative examples of lesions missed during AI‐assisted reading. (a) Ulcer with incomplete visualization and surrounding bubbles. (b) Lymphangiectasis with adjacent debris and bubbles. (c) Bleeding with surrounding bubbles and an overall darkened appearance. (d) Venous lesion partially visualized within the frame. [file DEO2-7-e70346-s002.zip › deo270346-sup-0002-SuppMat/deo270346-sup-0012-figureS3d.tiff]
